# 

**Published:** 1911-05-27

**Authors:** 


### BUILDINGS CONTEMPLATED AND IN PROGRESS.

London.—Mr. Edwin T. Hall, F.R.I.B.A., is architect for the new nurses' home at the London Homœopathic Hospital in Great Ormond Street. It contains separate bedrooms for seventy nurses. Messrs. Prestige and Co. are the contractors.

Brandon (Durham).—An isolation hospital is proposed to contain twenty beds. Particulars may be had from the Surveyor to the Urban District Council, Mr. G. G. Donkin.

Biggleswade.—Mr. Walter Jackson is architect for the proposed alterations to the Union Workhouse Infirmary. Tenders are invited and are to be delivered not later than June 6.

Cranbrook.—Alterations are contemplated to the Cranbrook Infirmary; forms of tender may be had from Mr. C. Payne, Surveyor, Cranbrook, and are to be returned before May 30.

Cardiff.—At the Cardiff Infirmary there has just been opened a new gynæcological operating-theatre at a cost of £2,500.

East Molesey.—An isolation hospital has been erected at East Molesey from a design by Messrs. Foster, Lovell and Lodge at a cost of about £3,336. Messrs. Appleby and Sons were the contractors.

Ellesmere Port.—The cottage hospital movement in Ellesmere Port has received material encouragement in a site covering a large area and including a valuable frontage to the main Whitby Road. It is the gift of E. P. Jones, Esq., J.P. The buildings necessary would cost between £2,000 and £3,000, and require an annual income of £600.

Gloucester.—With reference to the proposed new infirmary the Board of Guardians have appointed a deputation to meet the Local Government Board upon the question.

Lurgan.—Messrs. Hobart and Heron, architects, of Belfast, have charge of the Union Workhouse at Lurgan, to cost about £2,000.

Newark.—The tenders have now been received for the isolation hospital referred to in our issue April 29, the one accepted amounting to £2,344, by Mr. W. Smith, Millgate, Newark.

Staverton.—In connection with the suggested isolation hospital, information will be given by the Daventry Rural District Council's Surveyor, Mr. J. B. Williams.

Truro.—Builders desirous of tendering for the proposed additions to the Royal Cornwall Infirmary at Truro may inspect the plans and specifications at the office of the Architect, Mr. Alfred Cornelius, M.S.A. Prices should be delivered before the end of the month.

Thirsk.—The new isolation hospital at Thirsk has just been opened with due formality at a total cost of about £1,500. Eight beds, in two wards, and administrative block have been erected.

Tulla.—An asylum is contemplated at Tulla to cost about £10,000. Particulars may be had from Mr. B. E. F. Sheehy, 57 George Street, Limerick.

### Gifts and Bequests.

The Dreadnought Seaman's Hospital has received an anonymous gift of £1,000 to endow a bed.

Mr. Samuel Folwell has sent a donation of £105 to the Leicester Infirmary in loving memory of his late wife.

Mr. Alexander MacRosty, of Esher, Surrey, has left £150 to the London Hospital and £150 to Guy's Hospital.

The Bradford Royal Infirmary has received £100 from the executors of the late Mr. Joseph Hill, Park Drive, Heaton, in payment of the legacy bequeathed by him.

The Hospital for Consumption and Diseases of the Chest, Brompton, has received 50 guineas from Sir John Miller and £50 from Mrs. Frederic A. Phillips.

The Merchant Taylors' Company has sent ten guineas to the After-Care Association for Poor Persons discharged recovered from Asylums for the Insane, Church House, Dean's Yard, Westminster.

Mr. James Edward Renshaw, of Poulton-le-Fylde, Lancs, cotton-waste spinner, has bequeathed £1,000 to the Bury Infirmary, £250 for a seaside convalescent home for use by poor children of Bury, and a further £250 for its maintenance.

Mrs. Flora Jones Curling, of Welshpool, has bequeathed £50 each to the London Orphan Asylum, Watford, the Home for Incurable Children, Maida Vale, the British Home for Incurables at Clapham, and to the Royal Hospital for Incurables.

The Throat Hospital, Golden Square, has received £300 from Mr. A. Mortimer Singer.

The Chelsea Hospital for Women has received £10 10s. from the Skinners' Company.

Dame Annie Sophia Morrison, of Leeds, has bequeathed £100 each to Leeds Infirmary and to the Anti-Vivisection Society.

The Goldsmiths' Company has made a special grant of £500 to Westminster Hospital towards the Electrical and Bacterio-Therapeutics Departments.

Colonel Francis Haygarth, formerly of the Scots Fusilier Guards, has left, subject to the life interest of his wife, £1,000 each to St. George's Hospital, the Great Northern Central Hospital, the Belgrave Hospital for Children, and King Edward's Hospital Fund for London; and £500 to the Westminster Hospital.

The Royal Dental Hospital, Leicester Square, has received £52 10s. from Mr. Howard Morley and £21 from the Skinners' Company. The Merchant Taylors' Company has sent a donation of ten guineas to the After-Care Association for Poor Persons Discharged Recovered from Asylums for the Insane, Church House, Westminster. The Middlesex Hospital Cancer Charity has received £100 and £50 respectively from the Drapers' Company and the Salters' Company, being annual grants towards the maintenance of the Cancer Research Department.

### APPOINTMENTS VACANT.

Full particulars of these vacancies can be obtained on reference to our advertisement columns:

Beckett Hospital, Barnsley.—Second House Surgeon.
Charing Cross Hospital.—Clinical Pathologist and Bacteriologist.
Coventry and Warwickshire Hospital.—Junior House Surgeon.
Leeds General Infirmary.—Ophthalmic House Surgeon, Obstetric Officer, and House Physician.
Leicester Infirmary.—Senior House Surgeon and House Surgeon.
Prince of Wales's General Hospital, Tottenham.—Honorary Physician and Junior House Physician.
Queen's Hospital for Children, Hackney Road.—House Surgeon.
Royal Albert Hospital, Devonport.—Assistant House Surgeon.
Wolverhampton and Staffordshire General Hospital.—House Surgeon.

### COMING EVENTS OF THE WEEK.

**[Communications for this column must be sent to 29 Southampton Street, Strand, before noon on Wednesday.]**

Saturday, May 27.—Royal Caledonian Asylum. Anniversary dinner, Lord Lovat presiding, Hôtel Métropole.

Tuesday, May 30.—St. Pancras School for Mothers, 37 Charlton Street, N.W. Annual Meeting, Lady Carl Meyer in the chair, 3 p.m.

National Hospital, Queen Square, W.C. Lecture, "Polio-encephalo-myelitis," by Dr. F. Batten, 3.30 p.m.

Printers' Pension, Almshouse, and Orphan Asylum Corporation. Anniversary Dinner, Connaught Rooms, Great Queen Street, 6.30 p.m.

Wednesday, May 31.—Polyclinic, Chenies Street. Surgical Clinique by Mr. S. M. Smith, 4 p.m. Lecture, "The Value of the Radical Cure of Inguinal Hernia," by Mr. W. H. Battle, 5.15 p.m.

Convalescent Homes Association, 32 Sackville Street, W. Sixth Annual Meeting to be held in Committee Room, Sir Wm. S. Church in the chair, 4.30 p.m.

Thursday, June 1.—Polyclinic, Chenies Street. Medical Clinique by Dr. L. Guthrie, 4 p.m. Lecture, "Eclampsia," by Dr. T. G. Stephens, 5.15 p.m.

Friday, June 2.—National Hospital, Queen's Square, W.C. Lecture, "Traumatic Neurasthenia, by Dr. T. G. Stewart, 3.30 p.m.

Polyclinic, Chenies Street. Throat, Nose, and Ear Clinique by Dr. D. Mackenzie, 4 p.m.

London Homœopathic Hospital, Great Ormond Street, W.C.* Lecture, "Cases Illustrating the Selection of the Remedy by use of the Repertory," by John Weir, 5 p.m.

